# Neurotoxic Effects of Platinum Compounds: Studies *in vivo* on Intracellular Calcium Homeostasis in the Immature Central Nervous System

**DOI:** 10.3390/toxics3020224

**Published:** 2015-06-19

**Authors:** Graziella Bernocchi, Francesco P. Fanizzi, Sandra A. De Pascali, Valeria M. Piccolini, Caterina Gasperini, Violetta Insolia, Maria Grazia Bottone

**Affiliations:** 1 Dipartimento di Biologia e Biotecnologie “L. Spallanzani” Università di Pavia, via Ferrata 9, 27100 Pavia, Italy; E-Mails: valeria.piccolini@unipv.it (V.M.P.); caterina.gasperini01@ateneopv.it (C.G.); violetta.insolia01@ateneopv.it (V.I.); bottone@unipv.it (M.G.B.); 2 Dipartimento di Scienze e Tecnologie Biologiche e Ambientali (Di.S.Te.B.A.), Università del Salento, via provinciale Lecce-Monteroni centro Ecotekne, 73100 Lecce, Italy; E-Mails: fp.fanizzi@unisalento.it (F.P.F.); sandra.depascali@unisalento.it (S.A.P.)

**Keywords:** platinum compounds, neurotoxicity, CNS development, calcium homeostasis systems, purkinje neurons, dentate granule cells

## Abstract

Platinum compounds cause significant clinical neurotoxicity. Several studies highlight neurological complications especially in paediatric oncology patients with Central Nervous System (CNS) and non-CNS malignancies. To understand the toxicity mechanisms of platinum drugs at cellular and molecular levels in the immature brain, which appears more vulnerable to injury than in the adult one, we compared the effects *in vivo* of the most used platinum compounds, *i.e.*, cisdichlorodiammineplatinum (cisplatin, cisPt), and the new [Pt(*O*,*O*′-acac)(γ-acac)(DMS)] (PtAcacDMS). As models of developing brain areas, we have chosen the cerebellum and hippocampus dentate gyrus. Both areas show the neurogenesis events, from proliferation to differentiation and synaptogenesis, and therefore allow comparing the action of platinum compounds with DNA and non-DNA targets. Here, we focused on the changes in the intracellular calcium homeostasis within CNS architecture, using two immunohistochemical markers, the calcium buffer protein Calbindin and Plasma Membrane Calcium ATPase. From the comparison of the cisPt and PtAcacDMS effects, it emerges how essential the equilibrium and synergy between CB and PMCA1 is or how important the presence of at least one of them is to warrant the morphology and function of nervous tissue and limit neuroarchitecture damages, depending on the peculiar and intrinsic properties of the developing CNS areas.

## 1. Introduction

### 1.1. State of Art on Toxicity of Platinum Compounds and Aim

Platinating compounds have been recognized for over 30 years as anticancer agents used for the treatment of several types of tumors. Cisplatin (cis-dichlorodiammineplatinum II, cisPt) in particular, when introduced into clinical trials, has had a great impact in cancer medicine. Its clinical benefits were addressed to testicular, ovarian, cervical head and neck, non-small cell lung, and lymphoma cancers [1,2,3]. The discovery of cisPt was therefore a corner stone [4], which triggered great interest in platinum (II) derivatives. Besides cisPt, several platinum compounds were tested in clinical trials: The different platin compounds are used for different types of cancers because each varies in its efficiency for a specific tumor. For instance, it has been demonstrated in chemotherapies that cisplatin is superior to carboplatin in treatment of tumor testis, bladder, head and neck, small cell lung cancer and in paediatric malignancies whereas in other cancer types, carboplatin has tended to replace cisplatin. On the other hand, the use of platinum compounds is limited due to toxic side effects in normal tissues.

Toxicities associated with cisPt, that remains the most potent chemotherapeutic drug, range from mild to severe, being the most serious ototoxicity, nephrotoxicity, peripheral neurotoxicity and nausea and vomiting [3,5,6]. The benefit of cisPt is limited not only to neurotoxicity but also to resistance ascribable to the fact that cisPt is a multifactorial agent. In fact, its biochemical mechanism is based on the interaction of more intercellular changes [7], such as reduced drug intracellular transport, activation of oncogenes, inactivation of tumor suppressor genes, alterations in the cell cycle and checkpoints, and in apoptotic pathways, enhanced intracellular detoxification mediated by glutathione S-transferase (GST), and increased DNA repair or insensitivity to apoptotic cell death [8,9,10].

With regard to neurotoxicity, peripheral sensory neuropathy (PNS) is commonly observed in 50% of patients treated with cisPt [11,12], being the spinal dorsal root ganglia the primary location of cisPt damage [3]. Severely affected patients have sensory ataxia, but minimal or absent motor involvement [1]. In addition, cognitive deficits are a significant clinical problem after treatment with chemotherapeutic drugs that do not exclude persistent memory problems [13,14]. The deficits might be related to damages to central nervous system (CNS). A number of animal studies have shown that cisPt and its analogs affect several neurobiological processes [15], although the presence of mature brain blood barrier (BBB) would preserve from the cytotoxic effects since platinum-based agents do not have the propensity to enter the brain but only the dorsal root ganglia and peripheral nerves [16]. However, studies show that cisPt treatment is associated with increased BBB permeability, which further facilitates the passage of it across the barrier [17]. Neural and vascular lesions produced by cisPt are shown as hemorrhagic foci accompanied by necrosis and edema [18]. Neurotoxic effects on mature neurons were described in the cerebellar cortex and ventral horn of adult rats [19]. In addition, cisPt comes in contact with normal neuronal cells, such as stem or immature cells in the hippocampus [20], where long exposures to cisPt injured dendritic branches and reduced dendritic complexity that might contribute to cognitive impairment [21].

Neurotoxicity in the CNS should be seen as among the most serious problems to be addressed for tumors chemotherapy in infancy. Platinum compounds are widely used for childhood malignancies since they are essential components of multidrug frontline therapy regimens [22] given to children with CNS solid tumors (e.g., neuroblastoma) and non-CNS malignancies (e.g., leukemia) [23]. It is important to note that there are several long-term behavioural effects (such as depression, anxiety or antisocial behavior) in pediatric patients with cancer [24]. On the other hand, the immature CNS is more vulnerable to damage from toxic agents than the adult one and prone to impairment [25,26,27]. In mammals, there are crucial phases of CNS maturation during which the brain regions may have differential effects induced by various chemicals [25,28,29,30].

Based on the last remarks, in our research, we followed two goals: First, the study of *in situ* damages induced by platinum compounds on normal and immature neuroarchitecture, by means of morphological and molecular markers; second, the *in vivo* analysis of neurotoxic effects on normal tissue of a new platinum compound [Pt(*O*,*O'*-acac)(γ-acac)(DMS)] (PtAcacDMS) that encompasses the chemoresistance in comparison with cisPt.

### 1.2. An Old Platinum Compound vs. a New Platinum Compound. CisPt vs. PtAcacDMS: Action Mechanisms and Cytotoxicity

The main cellular target of cisPt is genomic DNA [31], although the full understanding of the process that translate DNA damages into its drug-mediated cellular effects is very critical. CisPt is a square planar neutral inorganic complex that reacts with DNA; for this interaction, the neutral cisPt has to be activated through spontaneous aquation reactions that make it strongly reactive. The cytotoxicity is first ascribed to its interaction with endogenous nucleophiles of purine bases to form interstrand and intrastrand DNA–protein and DNA–DNA crosslinks. The intrastrand DNA adducts are the main factor responsible for the cytotoxic action [2,32], which results in several biological effects, not necessarily directly correlated with cell death. Pro-survival and pro-apoptotic signals such as Bcl2 and Bax proteins are activated after cisPt exposure [2]. Nevertheless, cisPt is considered a very potent apoptosis inducer [33,34]. However, in the cytoplasm many cellular components with soft nucleophilic sites, such as cytoskeleton microfilaments, thiol-containing peptides and protein and RNA, may react with cisPt, being the most important non-DNA target glutathione (GSH) and thiol-containing biomolecules [32].

On the other hand, resistances to cisPt have been observed especially when in therapy protocols the standard clinical dose is doubled or chronic drug exposure is made. A number of cytoplasmic constituents inactivate cisPt. The concentrations of thiol-containing molecules increase after chronic cisPt administration, and induce resistance by decreasing the availability of the antitumor agent to interact with the target DNA. Increase in GSH has been shown in a number of cisPt-resistant tumor models and in the clinical studies [2,3]. Finally, increased repair of platinum-DNA adducts by several types of proteins, increased tolerance of cisPt adducts and failure of cell death are to be considered in the multifactorial resistance of cisPt [32].

Among the approaches to overcome toxicity and resistance linked to the clinical use of cisplatin, the synthesis of new platinum compounds having different target and a low toxicity profile should be highlighted. A class of new platinum complexes, specifically designed and synthesized by some of us, have shown some interesting biological properties; these complexes contain acetylacetonate (Acac) and sulfur ligands such as dimethylsulphoxide (DMSO) or dimethylsulphide (DMS) in the platinum coordination sphere [35,36]. The complex [Pt(*O*,*O*′-acac)(γ-acac)(DMS)] **(**PtAcacDMS) contains two Acac (one *O*,*O*-chelate, one sigma linked by methine in gamma position) and dimethylsulphide (DMS) in the metal coordination sphere. It was able to induce apoptosis in endometrial cancer cells (HeLa). In particular, its activity was about 100 times higher compared to the one of cisPt; moreover, PtAcacDMS showed an increased cytotoxicity in MCF-7 breast cancer cells that were resistant to cisPt [37,38]. This cytotoxicity concerned only an intracellular accumulation [37,38]. The reactivity, low with nucleobases and specific with sulfur ligands, suggests that the possible cellular target could be represented by aminoacid residues of proteins and enzymes involved in the pathway of apoptotic induction. In addition, mutagenic tests were performed on PtAcacDMS using cisPt as a positive control. Notably, whereas cisPt exhibits the renowned mutagenic activity, the new complex does not show revertant colonies. All these evidences together with intracellular signal transduction studies confirmed the biological activity of PtAcacDMS as the reaction with non-genomic biological targets [39], highlighting that DNA is not the main target of Pt(II) complex.

The new platinum compound [35,36] has been studied in MCF-7 cells [37,38,40], cells that are known to be resistant to many chemotherapeutics. It was confirmed that, compared with cisPt, the cytotoxicity of PtAcacDMS depends on its cell concentration in the tumour cells, not on its binding with DNA.

The presence of platinum in the developing brain tissue after PtAcacDMS administration [41] is in accordance with data obtained on HeLa and MCF-7 cells from *in vitro* studies [37,38]. Measurements of cellular accumulation of the novel cisPt analog is linearly correlated with its concentration, as it occurs in cisPt treated cells. PtAcacDMS concentration increases rapidly in the cells, and its cellular accumulation rate is calculated to be between 6 and 10 times higher than the cisPt one in HeLa and MCF-7 cells [37,38]. The cytostatic effect is closely related to the platinum accumulation in the cell. This issue could be an advantage for PtAcacDMS since it would permit the use of lower doses with consequent reduction of side effects and drug resistance risks.

In this article, the attention has been paid to the action of platinum compounds on intracellular calcium homeostasis [42]. To this aim we reported findings on the immunohistochemical changes of calcium protein markers in differentiating neurons which have a fundamental role for the neuroarchitecture and neural circuits’ establishment during postnatal life development.

### 1.3. Immature CNS Areas in the Postnatal Life of Mammals

Development of CNS in mammals does not end at birth; in fact, it continues through the postnatal life. Various morphological and physiological changes occur also in the human brain during infancy and adolescence [27,43]. The rate of developmental changes are very high in the infancy, whereas adolescence—which is the transition stage between childhood and adulthood and represents one of the most dynamic events of growth and development in the human—as a second surge of synaptogenesis still occurs. In these stages, developmental injuries and infections can result in several lasting CNS problems such as mental retardation, hydrocephalus and epilepsy. Neurotoxic agents including drugs and chemicals affect all steps of CNS development [25,28,29,30].

As models of postnatally developing CNS areas, the cerebellum [44,45] and hippocampal dentate gyrus (DG) [46,47,48] are brain regions particularly vulnerable in the developing animal as well as in newborn human because injury may alter physiology and cause neuropsychiatric disorders [49,50].

In the rat or mouse, both areas are later-developing brain regions with important neurodevelopmental changes [46,47,51,52,53], especially in the first three weeks of postnatal life when multiple interrelated processes occur in maturing neuroarchitecture. These range from cell proliferation, migration and differentiation to synaptogenesis and synaptic connectivity. Interestingly, the developing regions offer the opportunity to distinguish the events on which the platinum compounds act on DNA and non-DNA targets.

Considering the cerebellum, it is proved to be also a valuable model for studying naturally occurring cell death [54,55] *in vivo*. In the newborn, a germinative matrix—the external granular layer (EGL)—persists in the cerebellar cortex until the third week [52]. The matrix originates the granule cells [56] of only the internal granular layer (IGL), after their migration by Bergmann radial glia fibres. It is noteworthy that cerebellar defects were also found due to a failure of Bergmann radial glia, which is demonstrated to be a mechanical force of cerebellar foliation [57,58] in the maturation of the cerebellum architecture. The differentiation of the GABAergic Purkinje neurons even occurs postnatally, and it is regulated by the developing fiber systems in the cerebellum [52].

In the postnatally developing hippocampus, cell migration and differentiation continue to be very active. The critical steps in the histogenesis of rodent hippocampal formation have been described in detail [46,53,59]. While pyramidal cells and large interneurons arise during the prenatal life, DG granule cells originate at embryonic day E16 and continue to proliferate during postnatal development until they migrate to their final destination [51]. Therefore, cell migration and synaptogenesis are postnatal events of all cell types in the hippocampus [46,59].

Here, we focused the attention on cells that have a fundamental role in the neuroarchitecture and neural circuits’ establishment in the cerebellum and hippocampus, *i.e.*, Purkinje cells and DG granule cells, respectively. The Purkinje neurons are key elements being the sole neurons projecting outside the cerebellar cortex on deep nuclei in the white matter [60]; these large neurons extend their branched dendrite in the molecular layer of cerebellar cortex, where they receive synaptic contacts by parallel and climbing fibres, and by basket and stellate axons. The DG granule cells are cells whose dendrites penetrate in the molecular layer; here, they receive afferents mainly from enthorynal cortex, while their axons project outside the DG on pyramidal layer of Cornus Ammonis (CA) [46].

## 2. Experimental Section

### 2.1. Animals and Schedule of the Experimental Plan

The cisPt (Teva Pharma, Italy) and the new compound PtAcacDMS were administered to 10-day-old (PD10) Wistar male rats as previously reported [41,61].

The rats were exposed to a 12:12 h light:dark artificial cycle and were given *ad libitum* access to food and tap water. Treated and untreated control animals were anesthetized at PD11 and PD17 with the 35% chloral hydrate intraperitoneal injection (100 μL/100 gbw, provided by Sigma, St. Louis, MO, USA). Then, brains were quickly removed and processed as reported in Cerri *et al.*, [41]. Paraplast-embedded brains were cut in sagittal sections of cerebellar vermis and in cross sections of hippocampus.

All experimental protocol was in line with the Italian Ministry of Health (DDL 116/92) guidelines. All efforts were made in order to minimize the number of rats used and their suffering.

### 2.2. Immunocytochemistry in Light Microscopy

The sections were incubated with an endogenous peroxidases blocking buffer and then with the normal serum as described in Cerri *et al.* [41]. The presence of Calbindin (CB) and Plasma Membrane Calcium ATPase 1 (PMCA1) was detected through the use of a rabbit polyclonal anti-CB 28kD (diluted 1:2000; provided by Sigma, St. Louis, MO, USA) or a rabbit polyclonal anti-PMCA1 (diluted 1:1000; provided by Abcam, Cambridge, UK) in PBS. The sections were incubated overnight in a dark moist chamber with these antibodies. Thereafter, the immunocytochemistry signal detection was carried out as reported by Cerri *et al.* [41]. Then, an Olympus BX51 microscope was used, and the images were acquired with the digital camera Olympus Camedia C-5050 and then stored on a PC. Using Paint Shop Pro-7 (Jasc Software Inc., Eden Prairie, MN, USA), the images were corrected and converted in grayscale. The control brain sections were incubated overnight with PBS instead of primary antibody and no immunoreactivity was detected.

### 2.3. Immunocytochemistry in Fluorescence Microscopy

Localization of CB was achieved by using a mouse monoclonal anti-CB 28kD (diluted 1:5000, provided by Swant, Bellinzona, Switzerland) in PBS. The sections were incubated overnight in a dark moist chamber. Thereafter, the immunocytochemistry signal detection was carried out using Alexa Fluor 488-conjugated anti-mouse (diluted 1:100; provided by Molecular Probes, Space, Milano, Italy) as reported by Cerri *et al.* [41]. Then, an Olympus BX51 supplied with a 100 W mercury lamp was used with the following conditions: (i) 450–480 nm excf; (ii) 500 nm dm; (iii) 515 nm bf. Images were acquired with the digital camera Olympus Camedia C-5050 and then stored on a PC. Paint Shop Pro-7 (Jasc Software Inc., Eden Prairie, MN, USA, 2000) was used to adjust and convert the images in greyscale. The control brain sections were incubated overnight with PBS instead of primary antibody and no immunoreactivity was detected.

### 2.4. Determination of Cell Immunoreactivity Intensity

The optical density (OD) of CB and PMCA1 immunoreactivity was measured in the cerebellar Purkinje cell layer and DG granule cell layer. The extent of the labelling was determined on digitized images of sections acquired with an exposure time adjusted to avoid pixel saturation effect. The signal intensity was quantified by the means of densitometric analysis using Image-J 1.46p (Software NIH, Bethesda, MA, USA). Moreover, the mask shape was adjusted according to the spatial distribution of the cells within the layers. All the measurements were done using the mean value of the intensity over the area.

The immunostaining intensity for CB and PMCA1 were measured in a total of 30 fields (10 fields per rats) for untreated and treated animals, at PD11 and PD17. Data were recorded and analysed by Microsoft Office Excel Software spreadsheets and were indicated as the Means ± SD (standard deviation). The student’s *t*-test was performed to asses any statistical differences between control and cisPt or PtAcacDMS treated animals.

## 3. Results and Discussion

### 3.1. Platinum Compounds and Calcium Homeostasis

Despite the differences in the neurotoxic effects of the two platinum analogs, cisPt and PtAcacDMS, ascribable to a different mechanism of action, we can consider, as a common involved factor, the intracellular calcium concentration, that mediates the effectiveness and the toxicity of anticancer drugs. In fact, the modulation of intracellular calcium is a fundamental factor in the activation of cell death [42] or cell degeneration [62].

Overall, a central and critical role in neurogenetic events such as cellular proliferation, migration and differentiation, and synaptogenesis is plaid by calcium ions [63]. Disruption of function of calcium channels in signalling processes can lead to profound disturbances in brain cytoarchitecture and function. In particular, postnatal brain ontogenesis shows critical and vulnerable windows in which disturbances in calcium homeostasis should allow to dramatic consequences. In neurodegenerative mechanisms and neurological disorders, elevated levels of intracellular calcium are found [62]. On the other hand, elevated calcium transport controls the rate of cell migration, with spontaneous elevations in intracellular calcium levels [64].

Antitumor platinum compounds such as cisPt are often toxic through complex, not well understood mechanisms. CisPt may kill cells by various means such as apoptosis and necrosis. Perturbation of cellular calcium homeostasis [65] has been demonstrated in different *in vitro* models. Recently, Kawai *et al.* [66] showed in a non-tumour model, that relatively high concentrations of cisPt (250–750 μM) induce a rise in [Ca^2+^]_i_ while Liang and Huang [67] demonstrated in a cisPt-resistant cell line that [Ca^2+^]_i_ increases lesser and faster than in a cisPt-sensitive cell line. CisPt concentration-dependently increases [Ca^2+^]_i_ in HeLa-S3 (human cervix adenocarcinoma) and not in U2-OS (human osteosarcoma) cells [68]. One hypothesis regarding the interaction of cisPt and calcium homeostasis in HeLa-S3 cells is that cisPt might be actively transported into or out of the cell. When extracellular calcium enters the cytosol, an increase of [Ca^2+^]_i_ occurs and efflux transport mechanisms (Ca^2+^-ATPases) is activated, resulting in a consequent net calcium increase in calcium stores. Moreover, when the amount of calcium entering the cell and the efflux mechanisms are balanced, there is no further increase of [Ca^2+^]_i_ and the cytosolic concentration is higher than in control conditions. Calcium-dependent calpain is activated by an elevated [Ca^2+^]_i_, triggering apoptosis just in HeLa-S3 and not in U2-OS cells [4,61].

In summary, the expected cisPt related mechanisms of calcium intracellular concentration variations are: (a) cisPt, in a concentration dependent manner, causes a reduction of calcium current through the activation of calcium channels; (b) cisPt opens a membrane associated pore allowing calcium entry in the cells from the extracellular space, with a consequent increase in [Ca^2+^]_i_.

It should be mentioned that also inositol phosphate-3 (IP3) receptors are involved in the calcium entry [61].

CisPt affects calcium homeostasis by binding to sulphydryl groups of proteins such as enzymes, channels and pumps [69]; as above mentioned the binding of cisPt to non-genomic targets may contribute to its biochemical mechanisms of action [32].

PtAcacDMS affects free [Ca^2+^]_i_ through protein kinase C (PKC)-α-mediated closure of a few channels and the PMCA activity inhibition; the increase in [Ca^2+^]_i_ is related to its ability of triggering rapid apoptosis in MCF-7 cells [40].

Since our aim is the *in situ* correlation between calcium intracellular changes and neuroarchitecture damages, we considered specific histochemical markers of proteins involved in the calcium homeostasis.

### 3.2. Histochemical Detection of Calcium Homeostasis and Differentiating Cells in the Immature Cerebellum and Hippocampus

Intracellular calcium is regulated by delicate balance between the calcium entry and the active mechanisms involved in the transport against its concentration gradient. Pumps or exchangers of the outer cellular membrane regulate the calcium concentration; there is also a possible re-entry (via Ca^2+^-transport systems) to the calcium stores, mainly represented by mitochondria and endoplasmic reticulum. In the cell, calcium could also be bound to calcium-buffering proteins with consequent regulation of [Ca^2+^]_i_ [4]. Indeed, the association with various calcium binding proteins (CBPs) [70] can modulate intracellular calcium by a buffering action.

CBPs, including CB, parvalbumin (PV) and calretinin (CR), has been studied to identify different functional cell types in the brain neocortex, hippocampus, cerebellum and thalamus [71,72,73]. CBPs have been extensively studied as neuronal immunohistochemical markers of specific cell populations in the CNS [74,75,76] and have been implicated in neuroprotection in different pathological conditions since they function as buffers for calcium excess. Regarding the specific involvement, CB and PV sequester the excess calcium during development and synaptic plasticity. CBPs alter the duration of action potentials, and promote neuronal activity as protecting molecules against the damages derived from excessive calcium influx [74]. In the absence of CBPs, there is a high calcium accumulation inside the cytosol, causing hyperexcitability that often leads to neurodegeneration [71,72].

CBPs immunoreactivity has been detected in similar populations of neurons in mammals, such as humans, rodents and birds [77,78].

#### 3.2.1. Calbindin

This article focused on two cellular phenotypes, cerebellar Purkinje neurons and DG granule cells, both expressing one of the CBPs, the CB D-28kDa.

CB is a member of the EF-hand family of CBPs and it has a low molecular weight. CB is able to bind Ca^2+^ with a fast association rate [79]. CB seems to be involved in the modulation of specific calcium-dependent activities of developing neurons, such as neurite elongation and spine formation. CB inside the neuronal cytosol might exert a protective role against the overfull calcium influx, which may cause degenerative processes [80].

Developmental studies have indicated that CB is expressed early in embryonic CNS, following the cessation of mitosis, when neurons are ready to migrate [81]. CB has been shown to be temporarily expressed during brain development in numerous nerve cell systems and animal species [82,83].

In the rodent cerebellum [71,72], CB, CR as well as PV are detected in the cytosol of one or more populations of neurons at relatively high concentrations. The fast buffer CB is exclusively present in Purkinje cells, in the soma dendrite branches and axons. Neurons rich in CB appeared relatively resistant to degeneration in several acute or chronic disorders [72]. In the developing cerebellum, CB is low until embryonic day 18 and reaches a peak at postnatal day 20. Thereafter, the CB levels remain high [72].

**Figure 1:**
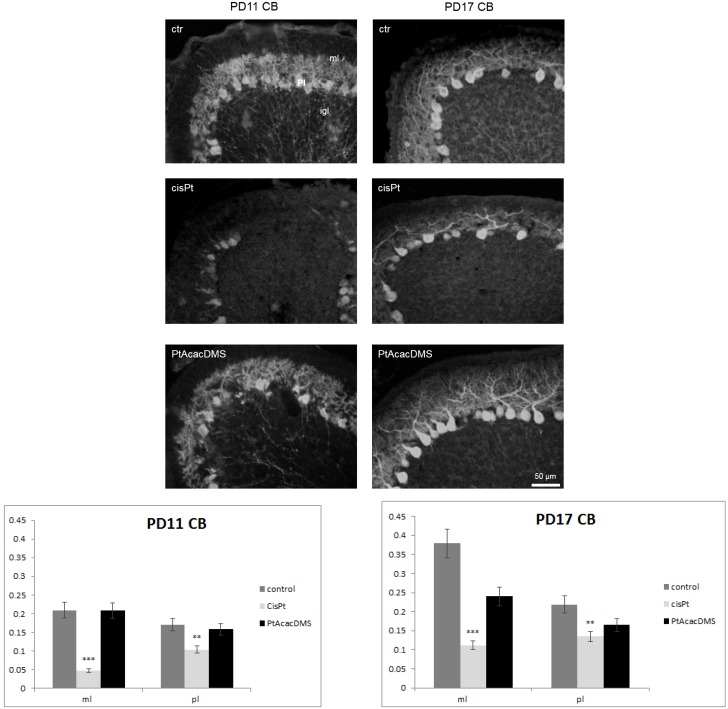
Calbindin immunocytochemistry in the Purkinje cell layer. At **PD11**, control rats (ctr) show intense labelling in the Purkinje cell cytoplasm; strong immunoreactivity is detected in the molecular layer, in the dendrite tree branches of Purkinje cells. After cisPt treatment, atrophy of Purkinje cell dendrite is observed as well as decreased intensity in the immunoreaction. After PtAcacDMS, Purkinje cells display the same intense labelling and dendrite morphology as in controls. At **PD17**, compared with controls, cisPt induces an evident decrease in the labelling of dendrite branches of Purkinje cells, while no changes are visible after PtAcacDMS. Histograms show the OD values and the significance of differences is reported (** *p* < 0.01; *** *p* < 0.001). ml: Molecular layer; igl: Internal granular layer; Pl: Purkinje cell layer. Scale bar: 50 μm.

At the postnatal stages of PD11-PD17 of control (ctr) rats (Figure 1), in the Purkinje cell layer, the large neurons were immunopositive to CB in all compartments, *i.e.*, in the soma and dendrite tree, and axon. Dendrite tree of Purkinje cells were labelled in the fine branches and spines.

There is good evidence of the presence of CB in Purkinje cell dendritic spines that may change their morphology to partially compensate for changes in Ca^2+^ homeostasis due to the loss of CPBs [71]. The intensity of immunolabelling increased at PD17 *vs.* PD11 (Figure 1), and the dendrite branches grew in the molecular layer (ML). In the ML, parallel and climbing fibres regulate the branching of Purkinje cell dendrite by synapses on its spines [84,85].

In the hippocampus, CB is primarily demonstrated in DG granule cell layer; the somata of granule cells are intensely immunostained while their dendrites are moderately stained. CB is also expressed in a CA1 pyramidal cell subpopulation and in some interneurons [59,74,75,86]. In particular, CB is present in GABAergic neurons mainly innervating the distal dendrite of the principal cells, and controlling the efficacy of afferent inputs. Hippocampal CB positive interneurons appeared resistant to epileptic injury in rat models [87].

Researchers have studied the ontogeny of cells which express CB at early postnatal stages [88,89], and in fact they showed that CB is present in granule cells of DG during the very early postnatal stages. CB has been reported to reappear on PD2 in clusters of neurons in the *radiatum* and the *oriens* layers and in the granule cells of the mouse DG [90].

**Figure 2:**
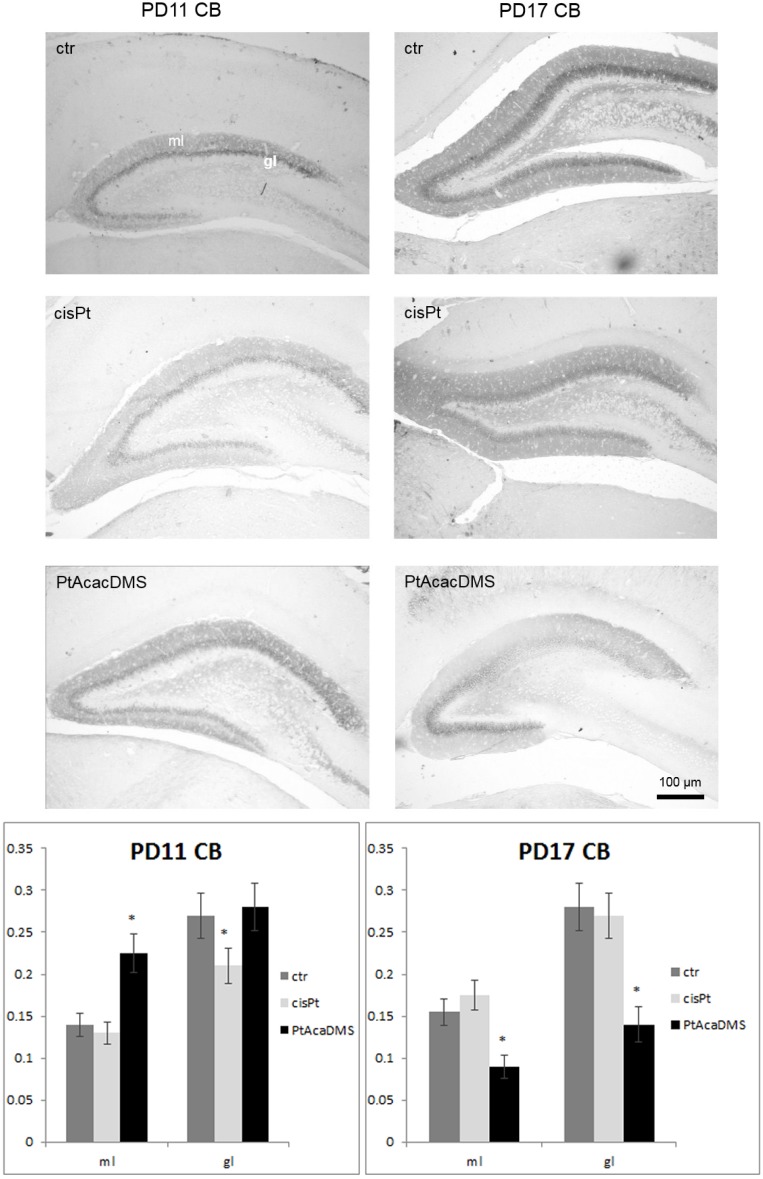
Calbindin immunocytochemistry in the DG granule cell layer*.* At **PD11**, the intensity of labelling in the DG granule cells decreases after cisPt and increases after PtAcacDMS. At **PD17**, decreased immunolabelling intensity is found after PtAcacDMS and not after cisPt. Histograms show the OD values and the significance of differences is reported (* *p* < 0.05). ml: Molecular layer; gl: Granule cell layer. Scale bar: 100 μm.

Progressive expression of CB appeared in an outside-in pattern in the DG granule cell layer as reported also by Abraham *et al.* [91] between PD11 and PD17 of control rat postnatal development (Figure 2).

Interneurons within the granular layer were also detectable as well as strongly CB labelled interneurons [48]. These latter were found at PD17 in the CA layers, where some groups of pyramidal cells were also stained. CB labelled fibers were also distributed in the ML and hilus of DG and beneath the CA3 pyramidal layer.

The intensity of labelling increased at PD17 *vs.* PD11. Changes in the immunolabelling to CB is indicative of changes in the level of the protein, since it has been shown that loss of CB after commissural kindling stimulation [92] is due to a decrease in the protein as shown by quantitative evaluations in granule cells of DG. The expression of CB is regulated by the functional state of the hippocampal circuit [79], since it is needed for normal short-term synaptic plasticity between the DG granule cells and CA3 neurons [93], and modulates the excitability of the CA1 pyramidal cells [94].

#### 3.2.2. Plasma Membrane Calcium ATPase

PMCA is a family of P-type Ca^2+^-ATPases found in the majority of eukaryotic cells [95]. PMCA is involved in maintaining a low cytosolic calcium concentration critical to neuronal function and survival; specifically, PMCA shows high affinity for calcium and acts by pumping this ion from the cytoplasm to the extracellular space [96].

In mammals, four PMCA isoforms (PMCA1-4) are expressed by four distinct genes, which generate over 20 variants by alternative splicing [95]. These isoforms show distinct activation kinetics and are distributed in a cell-specific manner [95,97]. All the PMCA isoforms are found in the adult brain, but not all of them are expressed at the same time, since they are related to specific cell types and to different maturation cell stages; this pattern of expression allows each neuron to control PMCA activity according to its calcium demands [98]. While little information is available on PMCA1, it is known that this isoform is expressed early in CNS development and is thus likely linked to synaptic maturation [99]. PMCA2 is induced on embryonic day E18 and attains high levels in the adult cerebellum [99,100]. The PMCA3 transcript appears during prenatal life and is generally upregulated in rodents during postnatal development [100]. An opposite profile is shown by PMCA4, *i.e.*, low expression throughout development and high levels in the adult nervous system [101].

PMCA activity is essential to avoid calcium overload that could be lethal to neurons [102], since calcium dysregulation is responsible for excitotoxicity, a process linked with neurodegeneration.

In the cerebellum [71], isoforms of Ca^2+^-ATPases (SERCAs) with different biophysical properties are detected in endoplasmic reticulum cisterns of Purkinje cells and are involved in the re-uptake of Ca^2+^, while Ca^2+^ is also extruded via plasma membrane Ca^2+^-ATPases (PMCAs) [103,104], which are localized in the Purkinje cell plasma membrane [105].

The best isoform that is particularly abundant in cerebellar Purkinje cells is PMCA2 [106,107,108], which is found in cell bodies and dendrites. While PMCA2 is highly expressed in the whole Purkinje cells, PMCA1 and PMCA3 appear to be restricted to the soma and dendrite branches, respectively, and these distributions are evolving according to cell maturation [109]. Moreover, PMCA1 is ubiquitously expressed from the earliest developmental stages [110].

Since we focused on cerebellum and hippocampus neurons containing CB, and on developing CNS stages, we have chosen a marker expressed by both types of cells, PMCA 1, the isoform labelling the extrusion of calcium from the cell.

**Figure 3:**
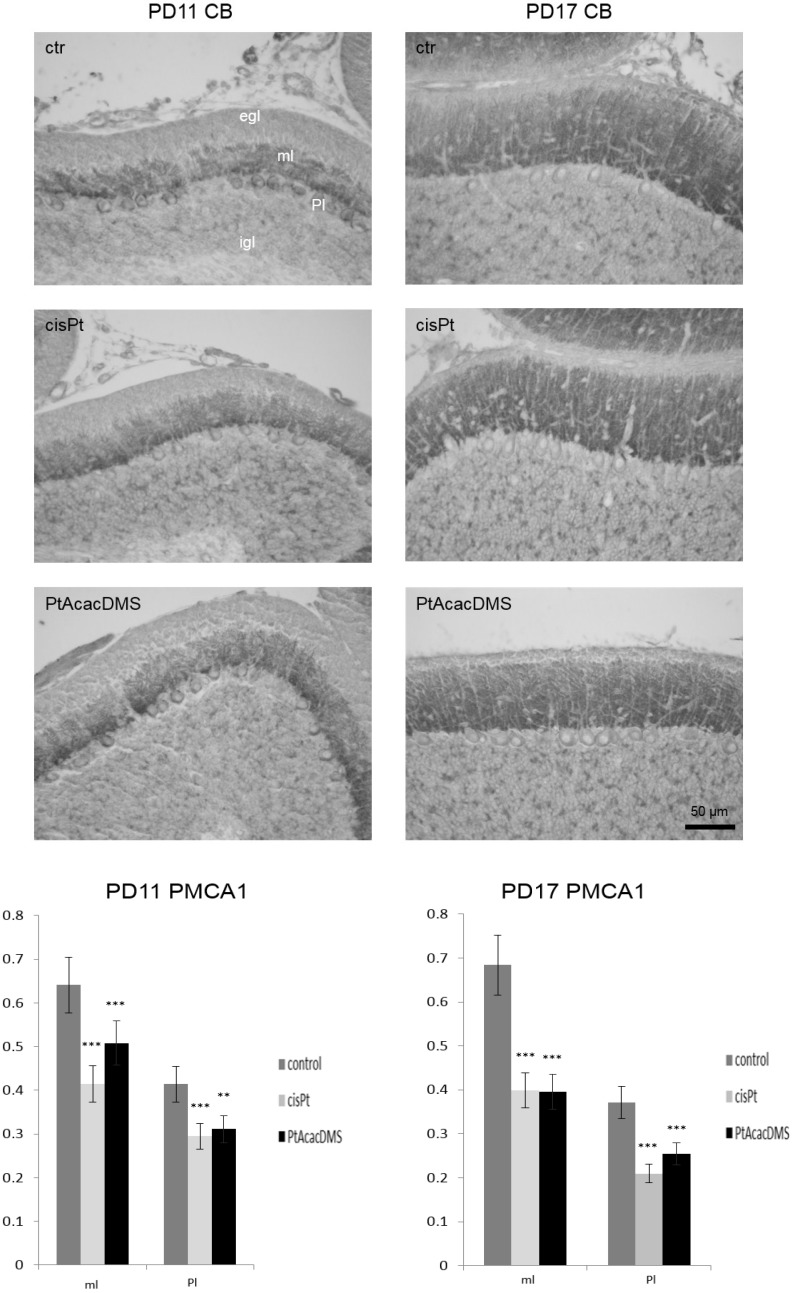
PMCA1 immunocytochemistry in the Purkinje neurons. At **PD11**, control rats (ctr) show intense labelling on the cell membrane of Purkinje cells; the cytoplasm is weakly positive while strong immunoreactivity is present in the molecular layer, in the Purkinje cell dendrite branches. After, cisPt and PtAcacDMS decreased labelling on the cell membrane and in the cytoplasm are observed, as well as in the molecular layer. At **PD17**, controls show a weak immunoreactivity *vs.* PD11; changes are observed after both platinum compounds treatment. Histograms show the OD values and the significance of differences is reported (** *p* < 0.01; *** *p* < 0.001). egl: External granular layer; ml: Molecular layer; igl: Internal granular layer; Pl: Purkinje cell layer. Scale bar: 50 μm

In the cerebellum (Figure 3), at PD11 control rats there was strong labelling for PMCA1 on plasma membrane of Purkinje cell soma, while weak positivity was shown inside the cytoplasm. The immunolabelling appeared to be present also on the growing dendrites. Instead, at PD17 there was a marked decrease in positivity in the Purkinje cell soma, whereas there was an increased labelling in the ML, likely due to synaptic contacts on dendrite branches. PMCA1 is one of the synaptic forms of the enzyme and, in fact, is being expressed as synaptic maturation progresses [111]. Our findings confirmed PMCA1 as a marker of immature Purkinje neurons and developing cerebellum [109].

In the hippocampus labelling, PMCA1 was detected in granule and pyramidal cell layers, and reflected the low density typical of plasma membranes in these layers, while intense punctate staining was detected in the neuropil [98,100,112].

**Figure 4:**
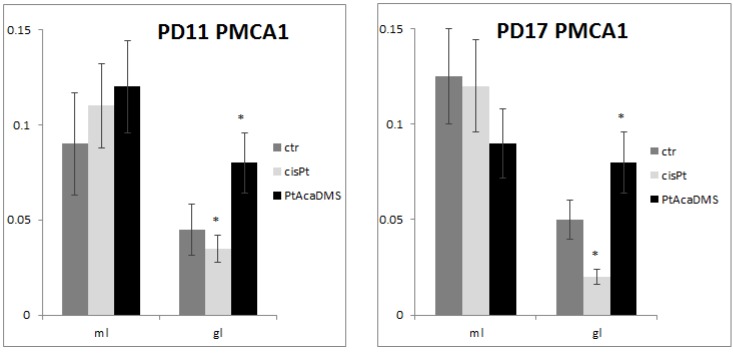
PMCA1 immunocytochemistry in the DG granule cell layer. Histograms show the OD values and the significance of differences is reported (* *p*<0.05). At **PD11**, the intensity of labelling decreases after cisPt and increases after PtAcacDMS. At **PD17**, decreased immunolabelling intensity is found after cisPt, while increased intensity is present after PtAcacDMS. ml: Molecular layer; gl: Granule layer.

Data obtained here indicated the presence of PMCA1 immunolabelling in the DG granule cells and molecular layer (Figure 4), both at PD11 and PD17; no changes in intensity of immunoreactions were detectable in the DG granule cells.

### 3.3. Platinum Compounds and Calcium Homeostasis in the Immature Neuroarchitecture

Table 1 and Table 2 summarize the trend of immunolabelling for CB and PMCA1 in the Purkinje and DG granule cell layers.

In the Purkinje cell layer, the treatment with cisPt caused at PD11 a strongly decreased CB immunoreactivity in the soma and dendrites, which appeared atrophic in several Purkinje neurons (Figure 1; Table 1). PMCA1 on the plasma membranes as well inside the cytoplasm markedly decreased (Figure 3). The decreased immunoreactivity of PMCA1 in the ML was localized in distinct puncta, likely due to synaptic contacts especially on Purkinje dendrite branches.

**Table 1:** Summary of the trends in the immunocytochemical indicators for CB and PMCA1 in the cerebellar Purkinje neurons with respect to controls following cisPt or PtAcacDMS treatment and the ensuing impact on [Ca^2+^]_i_.

Days of treatment	cisPt	PtAcacDMS
PD11	Decreased Ca^2+^ buffering: CB ↓	Steady Ca^2+^ buffering: CB =
	Decreased Ca^2+^ efflux: PMCA1 ↓	Decreased Ca^2+^ efflux: PMCA1 ↓
PD17	Decreased Ca^2+^ buffering: CB ↓	Steady Ca^2+^ buffering: CB =
	Decreased Ca^2+^ efflux: PMCA1 ↓	Decreased Ca^2+^ efflux: PMCA1 ↓

**Table 2:** Summary of the trends in the immunocytochemical indicators for CB and PMCA1 in the DG granule cells with respect to controls following cisPt or PtAcacDMS treatment and the ensuing impact on [Ca^2+^]_i_.

Days of treatment	cisPt	PtAcacDMS
PD11	Decreased Ca^2+^ buffering: CB ↓	Steady Ca^2+^ buffering: CB =
	Decreased Ca^2+^ efflux: PMCA1 ↓	Increased Ca^2+^ efflux: PMCA1 ↑
PD17	Steady Ca^2+^ buffering: CB =	Decreased Ca^2+^ buffering: CB ↓
	Decreased Ca^2+^ efflux: PMCA1 ↓	Increased Ca^2+^ efflux: PMCA1 ↑

Alteration of calcium homeostasis in the Purkinje neurons was described in mutants [71,72] and under experimental conditions in animal models as well as in human patients having neurological disorders. Notably, loss of CB (and PV) produces an alteration of the spine morphology which may be considered as compensatory mechanism. CB staining decreased in spinocerebellar ataxia and downregulation might cause alterations in the calcium homeostasis finally leading to Purkinje cell death. Moreover, the pivotal role of PMCA2 in the function of the cerebellum is shown by the phenotype of the PMCA2-null mouse and the deafwaddler 2 J, which is a mouse with a spontaneous mutation in the PMCA2 gene, and a consequent null pump activity. Both mice manifested cerebellar pathology consisting in motor deficit and ataxia [113]. In the cerebellum of PMCA2-null mouse, there was a reduction in the levels of mGlur1, which plays several essential roles in processes like motor coordination and associative learning. On the other hand, the dendrite branching of Purkinje cells as revealed by MAP2 immunocytochemistry was similar in wild type and knockout mouse; in addition, alterations in total and non-phosphorylated neurofilament-heavy (NF-H) immunoreactivity were shown [113]. These perturbations suggested altered formation of synaptic contacts, which might contribute to cerebellar dysfunction in absence of PMCA2.

On PD17, the strong loss of CB in the dendrite of Purkinje cells (Figure 1) was accompanied by a lower expression of PMCA1 in the soma (Figure 3). Thus, there was a down regulation of both calcium homeostasis systems.

After cisPt treatment, the early injury of both the systems regulating calcium homeostasis and their persistent imbalance in the most critical phase of Purkinje cell differentiation might alter deeply the growing of dendrite tree and synaptogenesis process. These alterations have been previously shown by the labelling for ionotropic and metabotropic glutamate receptors, and for the GABA enzyme GAD65 [84,85,114]. All these alterations are responsible for degeneration we observed several years ago in 20% of the Purkinje cell population [115].

In DG granule cell layer, the treatment with cisPt caused an early (one day after injection) decrease in CB immunoreactivity (Figure 2; Table 2). The loss of CB immunoreactivity was accompanied by decreased PMCA1 immunoreactivity (Figure 4). The loss and imbalance of calcium efflux and/or calcium buffering might be a possible or concomitant cause of cisPt neurotoxicity during DG postnatal maturation. Alteration of calcium homeostasis occurred in the superficial differentiating layers of DG and was accompanied by degeneration of mitotic granule cells that, on the contrary, are located in the deep granule cell layer [116]. Disturbance of the calcium ATPase pump in the DG persevered at PD17 (Figure 4), while CB immunoreactivity followed by recovery of calcium buffering (Figure 2).

The effects of PtAcacDMS on normal nerve cells differed from those of cisPt (Table 1 and Table 2).

At an early stage, at PD11, the intense immunolabelling for CB (Figure 1) showed an almost normal dendrite branching of Purkinje cells. In particular, the reaction intensity was the same as in controls. The expression of PMCA1 (Figure 3) in the soma and on the growing dendrite changed *versus* control rats; the immunoreaction intensity decreased. Later, at PD17, the PMCA1 (Figure 3) immunoreactivity was significantly lowered as compared with control rats, while no significant change in the CB labelling was found (Figure 1).

Therefore, PtAcacDMS treatment did not alter early the efficiency of both calcium homeostasis systems. The presence of the CB calcium buffering protein alone might guarantee the correct differentiation of Purkinje neurons [41,117] and the formation on synaptic contacts on them [118].

At PD11, as in the Purkinje cells CB expression was unchanged in the DG granule cells (Figure 2), which experienced an increase in the PMCA1 immunoreactivity (Figure 4). This indicates efficient calcium efflux from cells. At PD17, the decreased CB concentration and subsequent decreased calcium buffering was balanced by an increase in calcium efflux. Unequivocally, the balance involves a mechanism of calcium homeostasis in the DG.

### 3.4. Platinum Compounds and Morphological and Molecular Damages in the Immature Cerebellum and Hippocampus

Multiple acute effects of a single injection of cisPt have been demonstrated during the postnatally developing cerebellum. CisPt induced morphological and molecular changes, including the early damage of proliferating and postmitotic differentiating neurons. Apoptotic cell death in the EGL with concomitant alteration of migratory process, atrophy of Purkinje cell dendrite branches and formation of altered synaptic contacts were detected [114]. Our studies on the effects of cisPt on neurotransmitter molecules connected with the brain maturation, showed that neurotransmitters such as GABA, glutamate, and monoamines affected the cisPt-induced changes of developing cerebellum architecture [84,85,115,119], and therefore in the formation of synaptic contacts.

The whole cerebellar cortex presented numerous hemorrhagic foci, mainly seven days after the treatment that was carried out at postnatal day 10. Findings demonstrate that cisPt alters the blood vessels endothelium and could pass the BBB [115].

A recent report [116] has shown that cisPt alters the differentiation and maturation of synaptic contacts of some types of cells and interneurons of the rat hippocampus formation. These evidences help to explain its neurotoxicity on the developing brain.

The investigations on the toxic effects of PtAcacDMS on normal tissues pointed to a reduced neurotoxicity of the new platinum complex. PtAcacDMS induces less serious changes than cisPt on fundamental events in cerebellum and hippocampus development, such as no significant apoptotic events and less injured neuroarchitecture. In particular, the balance between Bcl2/Bax proteins [117] favours the PtAcacDMS treated rats, ensuring normal features in the highly actively proliferating cells in the cerebellum without cell death and cell migration [41]. The Bcl2 family proteins, which play a key role in regulating apoptotic cell death of many cell types [120] showed that after both platinum compounds treatments, Bax (pro-apoptotic protein) expression in Purkinje cells was more intense in respect to the controls. Nevertheless, the labelling for Bcl2 (anti-apoptotic protein) in the same neurons was lowered after cisPt treatment but maintained the steady state after the PtAcacDMS administration at lower dose and increased in the Purkinje cells at higher dose. Then, PtAcacDMS counterbalanced early the enhanced Bax immunoreactivity maintaining the Bcl2 control steady state, differently from cisPt. The balance might assure a limited cell death/cell degeneration in the Purkinje cell population [117].

The possible neuroprotective role of other specific cellular molecules, such as cellular prion (PrPc), has been studied [121]. After PtAcacDMS treatment, with respect to the controls, changes were detected in PrPc and apoptotic proteins. Instead, Bax immunopositive apoptotic bodies in the EGL were not detectable. The finding indicated that apoptotic cell death of proliferating granule cells is preserved. Co-localization of Bax and PrPc was clearly visible in the Purkinje cells; this may explain better the mechanisms through which PrPc and the apoptotic proteins cooperate. Based on the effects of both platinum compounds on Purkinje cell maturation, it should be emphasized that PrPc, supported by a synergistic action of the anti-apoptotic protein Bcl2, acts as a neuroprotective protein. Therefore, it counters the cytotoxicity in the postnatal critical phases of cerebellum development.

The mild cytotoxic effects of PtAcacDMS on the morphology of normal tissues may be due to the different subcellular targets of this compound. A greater efficiency of cell repair system, in the case of PtAcacDMS, to recognize the drug-target adducts and to repair them, may also be possible.

## 4. Conclusions

It is known that the favourable advantages and toxic effects of PtAcacDMS on endometrial cancer cells (HeLa) are due to a rapid and sustained apoptotic response characterized by (i) mitochondrial depolarization, (ii) cytosol accumulation of cytochrome c, (iii) translocation from cytosol to mitochondria of some proapototic proteins (the well-known Bax and the truncated form of Bid), (iv) activation of caspase 7 and 9, and v. chromatin condensation and DNA fragmentation [38]. In addition, data by Muscella *et al.* [40] on MCF-7 tumour cell lines showed that PtAcacDMS caused a decrease in the activity of PMCA (not SERCA or SPCA) and membrane permeability to calcium, resulting in the overall [Ca^2+^]_i_ increase. The effects of PtAcacDMS were also detectable when cells were stimulated with ATP; the decreased PMCA activity together with the closure of Ca^2+^ channels, opened by purinergic receptor, cause variations in Ca^2+^ level through the alteration of the purinergic system. Moreover, PtAcacDMS caused the activation of PKC-α and also the production of ROS that were involved in the calcium permeability changes and PMCA activity reduction. The overall effect of PtAcacDMS is to increase the [Ca^2+^]_i_, and may trigger rapid apoptosis in MCF-7 cells.

PtAcacDMS seemed to affect calcium homeostasis in the normal developing CNS differently than cisPt. Early, one day after treatment, although both platinum compounds affected PMCA activity, cisPt also actedon the CB buffering system. Instead, PtAcacDMS did not significantly affect the protein. Later, at seven days after treatment, the main difference between the two types of neurons is that, *in vivo*, the PMCA extruding calcium increased more in the DG granule cells in comparison with cisPt. Nevertheless, the Purkinje neurons conserve the capacity to cope with the calcium increase possibly induced by PtAcacDMS, although the calcium pump was lower in respect to controls. An intriguing hypothesis might be linked to multiple events having a role in the postnatal development of cerebellum and maturation of Purkinje neurons. All these events might be involved in the plasticity and recovery of cerebellar neuroarchitecture and function [84,85].

In conclusion, to cope with the excessive [Ca^2+^]_i_ which may injure neuron function and morphology, an efficient, rapidly-acting calcium buffering system or calcium pump shortly after treatment is essential.

Specifically, we have also demonstrated that the calcium pump may have an essential and a compensatory role when the buffering proteins are irrecoverable. At present, the evidence indicates the latter to be a possible mechanism to avoid calcium accumulation in nerve cells and thus prevent cell degeneration and death. The present study indeed emphasizes the need for further *in vivo* studies on the involvement of calcium homeostasis in the action of the platinum compounds. A deeper investigation on the different responses of cancer cells *versus* normal cells to platinum drugs is also required for a better understanding of their mechanisms of action.
